# Highlights from the IOB Conference on Cancer, 20-22 May 2021 and the new research vision of The Institute of Oncology Prof. Dr. Alexandru Trestioreanu, Bucharest, Romania.

**DOI:** 10.3332/ecancer.2021.ed114

**Published:** 2021-08-17

**Authors:** Simion Laurentiu, Dana Stanculeanu, Cristina M Capsa, Adina Stanciu, D Cristina Stefan

**Affiliations:** 1 University of Medicine and Pharmacy C Davila, Dionisie Lupu Street, no. 37, Sector 2, Bucharest, 4192910, Romania; 2 Institute of Oncology Prof. Dr. Alexandru Trestioreanu, Șoseaua Fundeni 252, Bucharest, 022328, Romania; 3 Department of Global Health, NUS-Duke University, 8 College Rd, Singapore 169857, Singapore

**Keywords:** Eastern Europe, oncology, Institute of Oncology, conference, Romania

## Abstract

The latest edition of the Bucharest Institute of Oncology (IOB) Conference was held between 20 and 22 May 2021, in the capital of Romania.

The conference was a hybrid event, with live and virtual participation and was attended by more than 1000 participants. The central theme was the value added by the tumor boards, underlining the significance of teamwork and collaboration between the different disciplines and oncology centers in the country.

The opening session was attended by representatives from national and international cancer associations and societies, the mayor of Bucharest, the Vice-chancellor of the University of Medicine and Pharmacy, C. Davila, the health advisor of the President of Romania, as well other dignitaries and health experts in oncology in Romania.

The conference sections covered all major types of cancer, education, research, health policies and new updates in guidelines and therapeutics.

Since it is a yearly event of the institute, the current situation of the institute was presented in the context of the full COVID-19 pandemic as well as the ways of managing and overcoming the negative impact on the access to care for cancer patients.

## Introduction

Romania has been affected by the COVID-19 pandemic for the last 18 months, a period of extraordinary efforts from all health sectors to contain the spread of the disease.

As the threat seems under control now, after two severe waves of infection and after long months of various disruptive restrictions, The Institute of Oncology in Bucharest decided to continue with the yearly tradition of holding an oncology conference, which is an opportunity for health professionals and all others involved in the care of oncology patients, to meet, discuss, present, and analyze the current situation in oncology and make plans and strategies for the future.

Founded in 1949, the Institute continues the tradition of specialized cancer care, initiated in Bucharest in 1926 at the Coltea Hospital, an establishment whose name was associated along the years with numerous accomplishments in Romanian medicine. In 1989, the Institute stepped onto a modern development path, becoming a powerful center of cancer diagnostic, therapy research and education in the country. Showcasing the progress achieved in controlling cancer at the Institute and in the country, the conference was also a stimulus towards new scientific and technical achievements.

## Conference summary

The conference, held in a hybrid manner, combined in-person presentations at Hotel Ramada Park in Bucharest with online contributions. It brought together more than 1000 delegates from the country and abroad, with an active participation of professionals from the diaspora. Clinicians, academics, researchers, Ministry of Health officials and representatives of various cancer institutions, congregated to share knowledge and adopt best practices in oncology.

The conference started on 20 May 2021 and lasted 3 full days with interactive sessions from 9 am until 20.40 pm every day in two concomitant locations. The scientific program included a variety of plenary sessions and discussion of clinical cases. The main theme was the value added by tumor boards, so real tumor boards were constituted for each session to discuss the presentations of several difficult cases, thus convincingly illustrating the benefits of this approach. The cases were followed by presentations of research or other projects.

Presentations were given across the oncological continuum of care. Strategies to help increase the vaccination rates against human papillomavirus infection (HPV), improve the early diagnosis of malignant diseases, increase the education of population, reinforce technical and medication resources and train health professionals by strengthening multidisciplinary oncology were all presented at this ambitious event. Several pharmaceutical companies sponsored various symposia offering insights into the value of their latest therapeutic products and approaches.

## Key conference themes

The key themes identified for the conference were as follows:

1. Breast cancer
2. Gastrointestinal cancers,
3. Gynecological cancers
4. Urological cancers
5. Lung cancers
6. Skin cancers
7. Research and projects at the institute

## Breast cancer

The session on breast cancer opened the conference, as the institute is recognized as a center of excellence in breast cancer and lung cancer. The tumor board analyzed and discussed several interesting and unusual cases such as breast cancer HER2 positive in pregnancy, orbital metastasis as initial symptom of breast cancer in a menopausal woman, advanced metastatic cases with unusual evolution and breast cancer in a male patient. The session also continued with the discussions of the surgeons related to assessing the sentinel nodes in patients who had neoadjuvant chemotherapy, prophylactic contralateral mastectomies, breast reconstruction, radiotherapy approaches aiming at limiting side effects and identifying prognostic factors in non-metastatic breast cancer by using the restricted mean survival time method.

**Figure 1.**
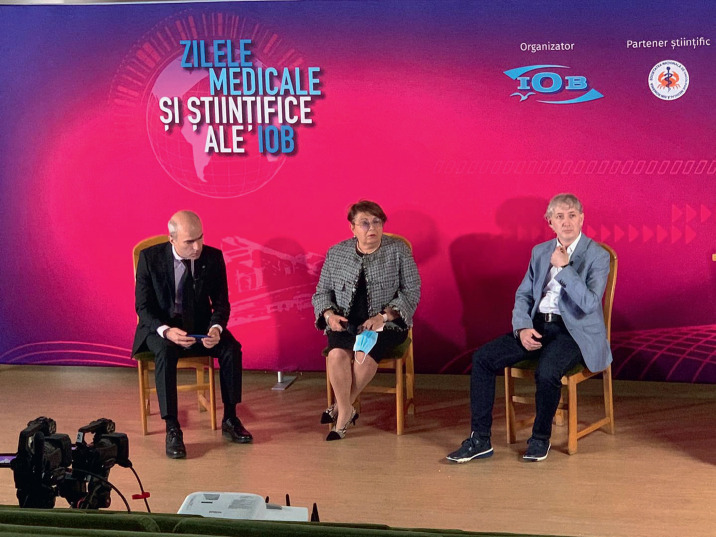
The opening of the conference

Professor Cristina Stefan gave an overview of the situation in breast cancer control. In Romania, breast cancer remains unfortunately one of the most common cancers for women and can be characterized as an unmatched burden. The country does not have a functional screening program and educational programs for the general population have limited coverage. There is a great difference between women living in urban areas versus those living rurally as well as between social classes. Globocan estimates that breast cancer is the 3^rd^ most common cancer in the Romanian population [1] (Fig 1). -Our country still lacks a national cancer registry.

The World Health Organization launched, on the 8th of March 2021, the 3rd global cancer initiative, this time aimed at breast cancer, with the objective of reducing mortality by 2.5% per year, averting 2.5 million breast cancer deaths globally between 2020 and 2040 [4, 5].

The 3 pillars of the WHO strategy – prevention, early diagnosis, and adequate treatment - were discussed and applied to local conditions such as the health promotion for early detection, timely breast cancer diagnosis and comprehensive breast cancer management.

**Figure 2.**
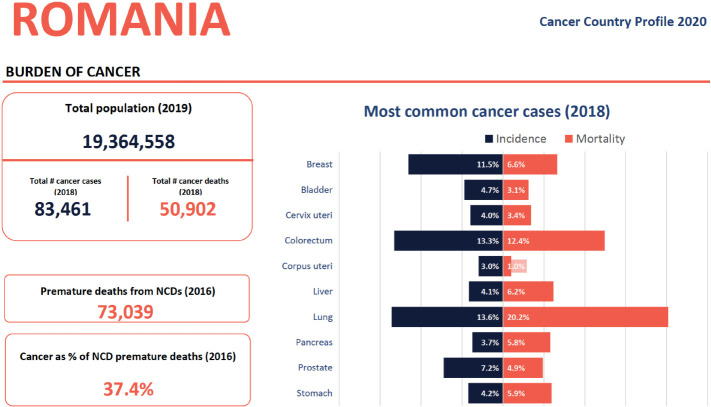
Breast cancer incidence and mortality (cancer country profile 2020, WHO) [2].

The capacity and quality of health systems at country level (Fig 2) needs to be improved by finalizing an operational and implementable national cancer control plan, a national registry and an NCD dedicated team part of the Minister of Health.

**Figure 3.**
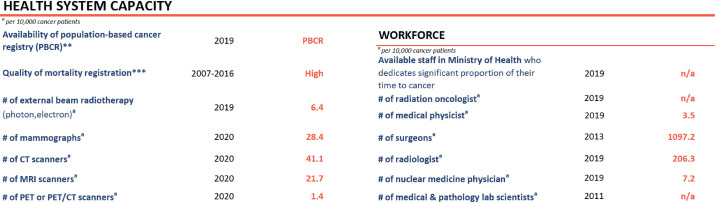
Health system capacity [2].

Pathology services should also be improved, telepathology introduced in the major oncology institutions in the country, palliation services be implemented across the country and a better balance between centralized and decentralized care should make it accessible for all patients. Fig 3 summarizes where Romania was last year in terms of formulating an effective coordinated response to cancer.

## Tumors of the digestive tract

The session attracted many speakers including Professor Irinel Popescu, from Fundeni Hospital, who pioneered the liver transplant in the country. The presentations included the liver transplant and the oncology therapy, the multimodal treatment of liver cancer, pancreatic and rectal cancer and prognos tic and predictive factors in colorectal cancer with metastases. Several unusual cases were presented, as well as surgical approaches, radiotherapy, and chemotherapy protocols.

One of the highly appreciated presentations discussed the role of artificial intelligence in taking therapeutic decisions for patients with colorectal cancers. The experience obtained in handling the association of SARS-CoV 2 infection and oncological treatment was also presented.

## Gynecological cancers

This session was one of the most popular, due to a large participation and interest from the participants. It included topics related to surgical challenges in vulvar cancer, advanced ovarian cancer and multimodality treatment, cases of oncological treatment for ovarian cancer in the context of hemodialysis, cervical cancer and HIV, conservation of fertility in the endometrial cancer, new treatment developments in advanced ovarian cancer, the need for accelerating the cervical cancer prevention, screening, and treatment program in the country.

### HPV vaccination in Romania

In Romania, cervical cancer is a public health issue, being the second most frequent type of cancer in women between 15 and 44 years old. Annually, 4000 women are diagnosed with this disease, of which 2000 will die [6].

Cervical cancer represents 12% of the total number of neoplasms diagnosed each year in the European Union, our country ranking first regarding incidence, which is three-fold higher than the European average (34.9/100,000 women in Romania versus 11.3/100,000 women in EU) [6, 7].

Romania also has the highest mortality due to cervical cancer, mortality of 16/100000 compared to an average of 6/100000 for other countries and a low rate of mortality below 2/100000 in France and Italy.

The vaccination campaign is addressed in the country to young girls between the age of 11 and 14 years old and until the end of April 2021 only 28.169 young girls were vaccinated, the initiative moving extremely slowly towards achieving the desired 90% of population vaccinated before 2030. There is at the moment no free vaccine for girls above the age of 15 years or for boys.

## Urological cancers

Besides the interesting case reports, this section included a presentation on renal cell carcinoma – Romanian landscape, a comprehensive review of the situation of this particular tumor and its control in the country.

## Various tumors

A few presentations covering pathology that was not discussed elsewhere in the conference fell under that category. Among them, we noted the correction signaled by Dr. Adina Stanciu, who observed that in diabetic patients with differentiated thyroid cancer undergoing radioiodine therapy, the cardiovascular risk evaluated by the Framingham score did not account for the reductions in the ejection fraction due to the radioactive therapy, while the HeartScore was more accurate in that respect.

## Lung cancer

The discussions were centered around the multidisciplinary teams involving the thoracic surgeon and the timely surgical interventions, the pathology of NSCLC stage 4, predictive biomarkers of response to immunotherapy in lung cancer, the perspective of the oncologist leading tumor boards, surgical thoracic wall reconstruction, new modern technologies supporting the young thoracic surgeon, gene alterations in advanced NSCLC, the association between radiotherapy and immunotherapy, all these amongst a number of unusual, difficult cases presentations.

## Skin cancers

Most of the presentations in this subsection were related to various aspects of diagnosing and managing malignant melanomas.

## Tumour boards

The Tumor board as a multidisciplinary decision team was introduced in Romania as compulsory by the Minister of Health in 2019. Despite the regulations, many oncology centers, public and private hospitals in the country do not still discuss collaboratively the best therapeutic approach for the patient. A large number of patients is using second clinical opinion or prefer to be treated in oncology centers overseas.

The Institute of Oncology in Bucharest recently introduced the regular multidisciplinary team discussion of the oncology patients creating at the same time the platform needed for other referrals and second opinion of patients from the other regions of the country.

## Impact of the pandemic on the IOB

The pandemic required the redirection of personnel and technical means towards performing the overwhelmingly large number of SARS-CoV 2 tests. Additional personnel were hired, and new sequencing equipment was brought in. Some research projects were delayed, and other projects were not started, waiting for better circumstances.

Most of the activity was centered around testing and treating for COVID-19 and basically every hospital and institute in the country was transformed into a unit of support for dealing with infectious emergencies and corresponding treatment.

Many projects and initiatives were related to acquisition of sequencers and providing facilities and creating opportunities for improving the infrastructure in order to respond to the present pandemic challenges.

The institute started testing for COVID-19 only in November but executed in less than 6 months already 14361 tests of which 491 were positive (3.4%)-92 medical personnel and 399 patients.

The institute performed between 2-300 tests a day, with a result in less than 5 hours.

The equipment acquired will, at the end of the pandemic, make it possible to initiate new projects, particularly in the field of precision medicine.

**The largest cancer institute in the country was affected as well by the adversities presented by the large number of infections, saturated health systems and overloaded hospitals. However despite a number of restrictions and change in prioritization such as limited screening, delayed routine surgery, prolonged admissions in the hospital, the institute of oncology benefited from a number of additional investments done for improving health care.**

**In the same context, now, as we are crossing a more stable period, a new medical and research strategy is being formulated and the institute is preparing for readiness in applying to be part as a strong collaborator of the numerous European research opportunities.**

## Research and future plans at the institute

**Figure 8.**
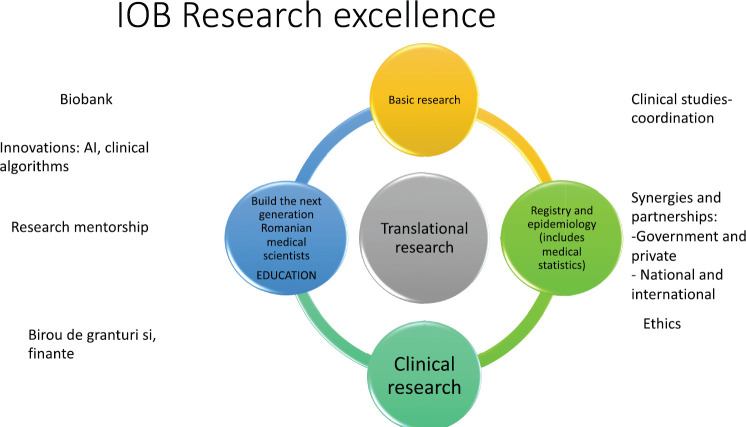
A comprehensive vision for the laboratory at the Bucharest Oncology Institute.

Research is under reorganization, with the ambition of creating a center of excellence in genomics, promoting research education and mentorship, collaboration, and partnerships locally and internationally (Fig 8).

## Conclusions

The conference at the largest institute of oncology in Romania, held between the 20th and 22nd of May 2021, enjoyed a large audience and shared a team spirit, reflected in the theme chosen: tumor board and the role of the interdisciplinary teams.

- A high number of unusual, difficult case presentations were discussed, illustrating the role of tumor boards.
- Projects and initiatives from the country, as well as expertise, were shared.
- In the context of the major cancer control initiatives launched by WHO recently, urgent efforts should be made to draft the national cancer plan.
- The conference was a broad overview of cancer research at the Institute and highlighted the need for planning more projects and initiatives at local and international level.
- The numerous attendants, predominantly young professionals, indicate that the potential for research in Romania is high and the return of the investment in equipment and training will be rewarding.

## Conflicts of interest

The authors declare that they have no conflicts of interest.

## Funding

No funding.

## Authors’ contributions

All authors participated at the conference with multiple presentations, organization and management, contributed with summaries of presentations.

## Ethics approval and consent to participate

Not applicable.

## Consent for publication

During the event, all the participants authorised the taking of photos, video and their publication at the time of registration in accordance with the current privacy protection policy. The publication of the remaining photographs has been authorised by the official services that are the owners of these photos, fulfilling what is established internally in its policies of protection and privacy.

## Figures and Tables

**Figure 4.**
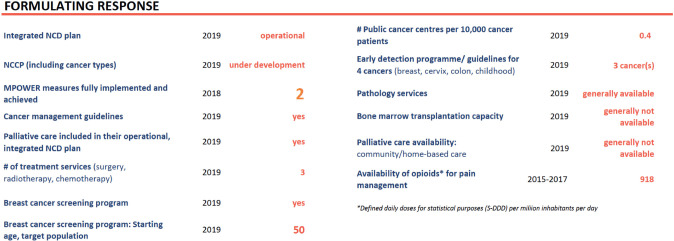
On the path of formulating a response to cancer, Romania 2020 [2].

**Figure 5.**
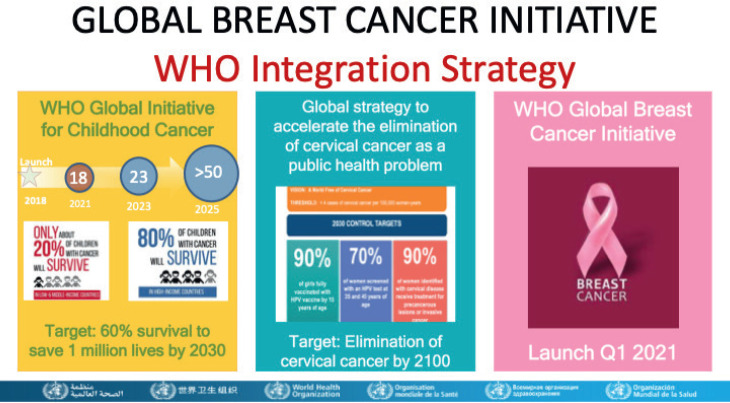
WHO proposed integration of services.

**Figure 6.**
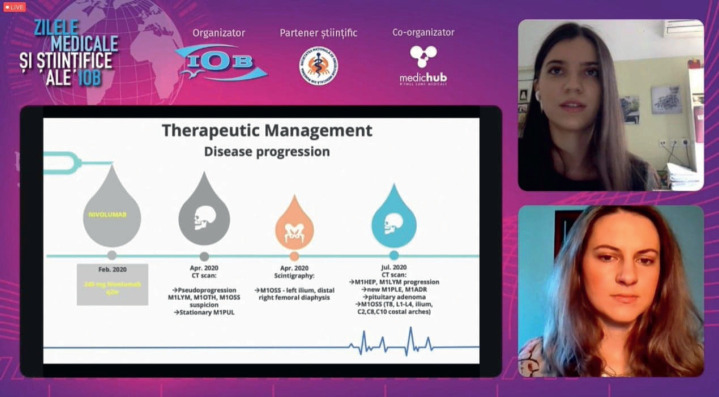
Treating renal cell carcinoma in a young adult: challenges and approaches

**Figure 7.**
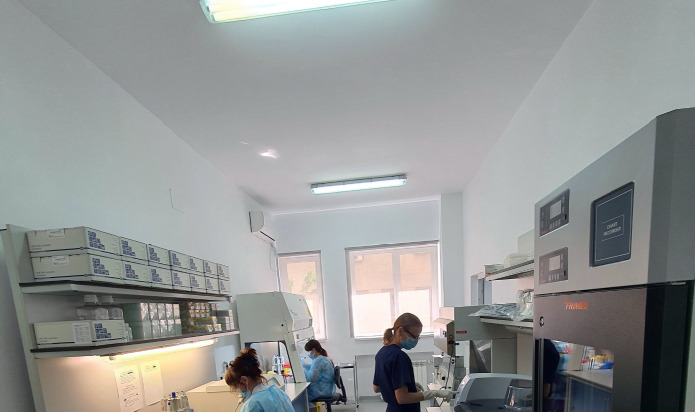
The modernized laboratory of molecular genetics
